# Antinuclear antibodies (ANA) in a Colombian cohort of pediatric patients with autoimmune diseases (PAID)

**DOI:** 10.1186/1546-0096-9-S1-P230

**Published:** 2011-09-14

**Authors:** MD Yepez Ricardo, MD Malagon Clara, Consuelo Romero-Sanchez, MD Mosquera Catalina

**Affiliations:** 1 Pediatric Rheumatology Fellowship program, Universidad el Bosque, Bogotá, Colombia; 2 Pediatric Rheumatology Fellowship program, Universidad el Bosque, Bogotá, Colombia; 3 Instituto UIBO, Universidad el Bosque, Bogotá, Colombia; 4 Pediatric Rheumatology Fellowship program, Universidad el Bosque, Bogotá, Colombia

## Background

ANA is an important diagnostic tool in PAID. The type of staining patterns by IFF technique and the final titer varies according of the type of PAID.

## Aim

To evaluate the ANA type of patterns and titer on a cohort of PAID patients in a referral pediatric rheumatology center in Bogotá.

## Methods

Retrospective descriptive study of a PAID cohort.

## Results

363 patients were included. 66% had JIA, 11% JSLE, 7% juvenile undifferentiated autoimmune disease(JUAD), 4% Scleroderma, 4% JDM, 1% uveitis ANA + and a group of other PAID 11%. ANA positivity varies according to the subtype of JIA from 0-68%. Positive AAN: JSLE 93%, scleroderma 85%, JDM 63%. Most JUAD had ANA+ at low titers. A heterogeneous group of other PAID showed a variable titers and patterns of ANA .The most prevalent type of pattern was speckled, followed by homogenous and other pattern were uncommon. On JSLE, the prevalence and titers were higher and multiple auto antibodies were detected at the time of diagnosis. (Table 1).

**Table 1:** 

Disease	N	ANA+	Speckled	Homogeneous	≤1:320	>1:320
JIA	98/240	41%	96%	4%	91%	9%

JSLE	37/40	93%	70%	24%	35%	65%

JUAD	24/24	100%	83%	13%	63%	37%

JDM	8/13	62%	62%	38%	88%	12%

Scleroderma	11/13	85%	82%	18%	55%	45%

Other PAID	24/31	71%	71%	21%	67%	33%

Uveitis ANA+	3/3	100%	100%	0%	100%	0%

## Conclusion

ANA positivity is not specific for PAID. Its interpretation should be based on a good clinical correlation and additional work up in order to classified and confirm PAID.

